# Association between socio-environmental factors, coverage by family health teams, and rainfall in the spatial distribution of Zika virus infection in the city of Rio de Janeiro, Brazil, in 2015 and 2016

**DOI:** 10.1186/s12889-021-11249-y

**Published:** 2021-06-23

**Authors:** Carlos Eduardo Raymundo, Roberto de Andrade Medronho

**Affiliations:** 1 Instituto de Estudos em Saúde Coletiva, Universidade Federal do Rio de Janeiro, Rio de Janeiro, State of Rio de Janeiro Brazil; 2 Present address: s/n - Próximo a Prefeitura Universitária da UFRJ Rio de Janeiro, Avenida Horácio Macedo, Rio de Janeiro, State of Rio de Janeiro 21941598 Brazil; 3 Faculdade de Medicina, Universidade Federal do Rio de Janeiro, Rio de Janeiro, State of Rio de Janeiro Brazil

**Keywords:** Zika virus, Spatial analysis, Socio-environmental, Arbovirus, Theoretical model

## Abstract

**Background:**

Zika virus (ZIKV) infection caused outbreak in Brazil, in 2015 and 2016. Disorganized urban growth, facilitates the concentration of numerous susceptible and infected individuals. It is useful to understand the mechanisms that can favor the increase in ZIKV incidence, such as areas with wide socioeconomic and environmental diversity. Therefore, the study analyzed the spatial distribution of ZIKV in the city of Rio de Janeiro, Brazil, in 2015 and 2016, and associations between the incidence per 1000 inhabitants and socio-environmental factors.

**Methods:**

The census tracts were used as the analytical units reported ZIKV cases among the city’s inhabitants. Local Empirical Bayesian method was used to control the incidence rates’ instability effect. The spatial autocorrelation was verified with Moran’s Index and local indicators of spatial association (LISA). Spearman correlation matrix was used to indicate possible collinearity. The Ordinary Least Squares (OLS), Spatial Lag Model (SAR), and Spatial Error Model (CAR) were used to analyze the relationship between ZIKV and socio-environmental factors.

**Results:**

The SAR model exhibited the best parameters: R^2^ = 0.44, Log-likelihood = − 7482, Akaike Information Criterion (AIC) = 14,980. In this model, mean income between 1 and 2 minimum wages was possible risk factors for Zika occurrence in the localities. Household conditions related to adequate water supply and the existence of public sewage disposal were associated with lower ZIKV cumulative incidence, suggesting possible protective factors against the occurrence of ZIKV in the localities. The presence of the Family Health Strategy in the census tracts was positively associated with the ZIKV cumulative incidence. However, the results show that mean income less than 1 minimum wage were negatively associated with higher ZIKV cumulative incidence.

**Conclusion:**

The results demonstrate the importance of socio-environmental variables in the dynamics of ZIKV transmission and the relevance for the development of control strategies.

**Supplementary Information:**

The online version contains supplementary material available at 10.1186/s12889-021-11249-y.

## Background

Zika virus (ZIKV) infection caused a major outbreak in the Americas, especially Brazil, in 2015 and 2016. In October 2015, Brazil reported to the World Health Organization an unusual increase in cases of microcephaly [1]. Evidence mounted for the association between ZIKV and microcephaly leading the WHO declared ZIKV a “Public Health Emergency of International Concern” in February 2016 [2–5].

However, in November 2016, the WHO declared that the ZIKV epidemic is no longer a “Public Health Emergency of International Concern”. The Brazilian government also closed down the ZIKV program as a “Public Health Emergency of National Concern”. Data in Brazil show that the number of probable cases decreased considerably since the announcement by the Ministry of Health: in 2016 there were 216,207 cases, dropping to 17,593 in 2017 and 2493 in 2018 [6–8]. In order to avoid new outbreaks of the disease, it is thus necessary to identify the risk factors for ZIKV. As with other arbovirus infections such as dengue (DENV) and chikungunya (CHIKV), the highest incidence of ZIKV also appears to affect areas with greater social inequality [9–13].

Disorganized urban growth, facilitates the concentration of numerous susceptible and infected individuals in the same geographic area [14, 15]. The localization and control of less socioeconomically favored areas can thus help identify possible mosquito breeding sites. Studies in the USA and United Kingdom have also shown that contextual socioeconomic factors influence the occurrence of infectious diseases [16]. Study about dengue virus, realized between the years 2008 and 2018, identified that areas of disorganized urban growth observed the highest incidence of dengue [17]. Other study in the city of Rio de Janeiro evaluated the incidence the dengue, zika and chikungunya in the poor community area in the years 2015 and 2016. The results found high spatial variability for three arboviruses [18].

Inter-annual variability in the climatic zones can influence the increase in the mosquito population and thus the growth in arbovirus cases. Historically, South America and West Central Africa were projected with the greatest increases in inter-annual variability [19]. In 2015, South America experienced the “El Niño” phenomenon, with periods of heavy rainfall. Studies indicate a possible association between “El Niño” and the Zika epidemic’s spatial spread [20–23]. The El Niño also associated with case occurrence and distribution for dengue and chikungunya [22, 24–27].

Since Zika is an asymptomatic disease in some cases, infected individuals do not always seek healthcare services. In addition inadequate knowledge on Zika [28], poor attitude towards Zika [29] and low awareness about Zika testing [30] among frontline healthcare workers also could contribute to underreported case of Zika. A study in French Polynesia, Martinique, and Guadalupe suggests that underreporting of cases can range from 3 to 50% [31]. However, locations in Brazil covered by the “Family Health Strategy” (FHS) may favor measures to fight and control the mosquito vector, besides an increase in active search for ZIKV cases. Healthcare workers under the FHS are trained to implement health education activities such as urban cleaning and basic sanitation [32, 33]. This comprehensive approach to health can thus favor greater adherence to health services, especially in more vulnerable populations [34].

The characterization of risk areas could contribute to decision-making during new outbreaks of ZIKV. In this scenario, statistical techniques for spatial analysis in health have been used to help determine environmental factors and epidemiological patterns [35].

It is thus useful to understand the mechanisms of health inequality that can favor the increase in ZIKV incidence, especially in areas with wide socioeconomic and environmental diversity. The city of Rio de Janeiro was selected for this purpose because of its large population, heavy socioeconomic imbalance in all areas of the city, and the presence of environmental protection areas such as forests, parks, and coastline. In addition, the state of Rio de Janeiro had the country’s third highest ZIKV incidence according to data from the Epidemiological Bulletin on Monitoring of Microcephaly Cases in Brazil in 2016 [36].

The study aimed to analyze the spatial distribution of ZIKV in the city of Rio de Janeiro in 2015 and 2016 and identify factors associated with the occurrence of ZIKV.

## Methods

### Study site

**Fig. 1:**
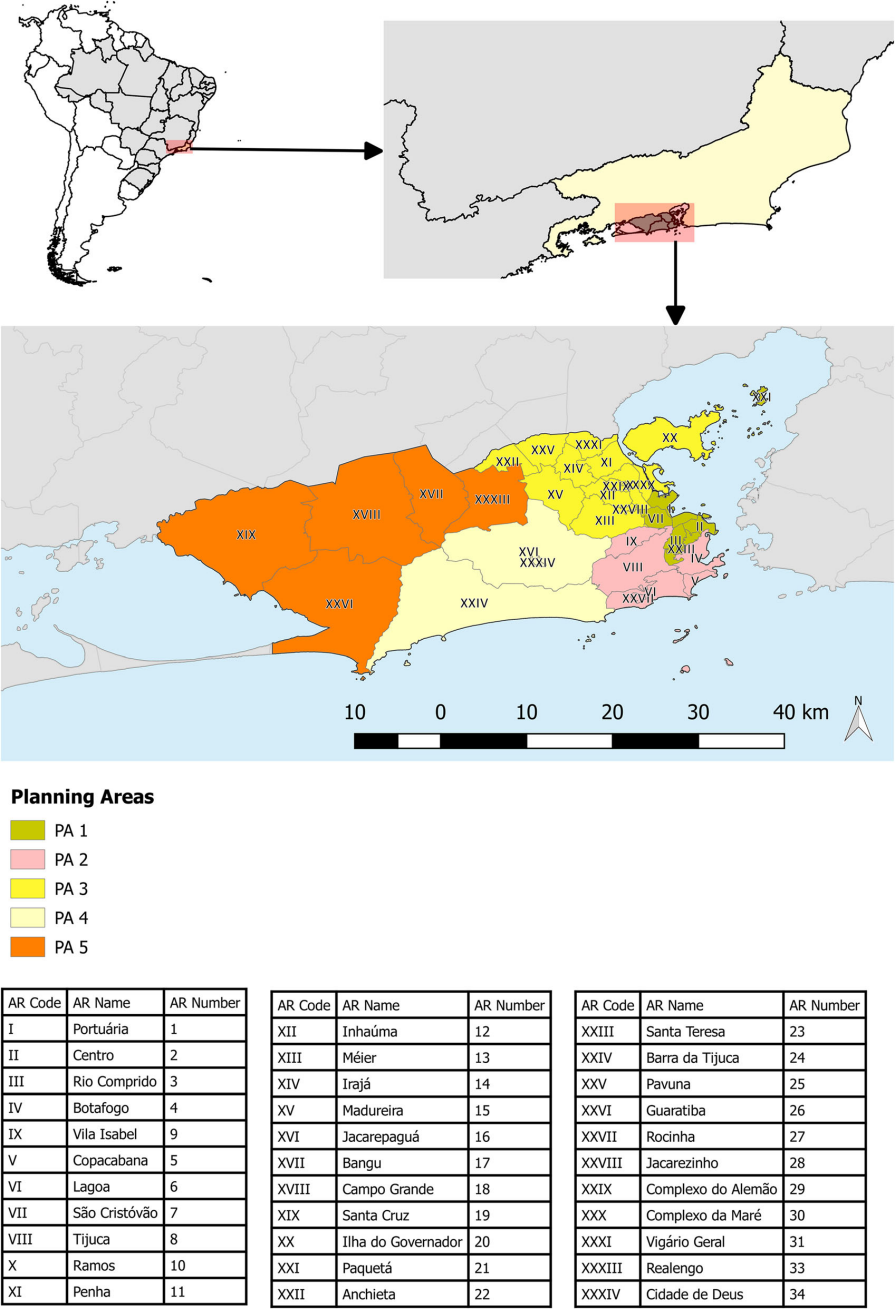
Geographic location and planning areas of the city of Rio de Janeiro. Map created in R software version 3.4.3 by authors. Source - Brazilian Institute of Geography and Statistics, and Pereira Passos Institute of the Rio de Janeiro Municipal Government [38, 39]

This is study conducted at the level of the census block in the city of Rio de Janeiro, located in the Southeast region of Brazil at latitude 22°44′45.59“S to 23°04’58.34”S and longitude 43°05′48.89“W to 43°47’43.79”W. The city has a population density of 5599.93 inhabitants/km^2^ and an exclusively urban population estimated at 6,718,903 inhabitants in the year 2019 [37]. The city is divided into 160 neighborhoods, 34 AR, 5 major PA. The city has 10,504 census tracts, with major socioeconomic differences distributed across all the regions. Approximately 22% live in substandard clusters or favelas [38]. Figure 1 shows the geographic location of the city of Rio de Janeiro and subdivision in five major Planning Areas and 34 Administrative Regions.

### Availability of data and materials

The analytical units in this study were the census tracts, based on data from the latest Population Census by the Brazilian Institute of Geography and Statistics (IBGE) in 2010. Secondary data were collected from three information sources for 2015 and 2016: (i) new cases daily of ZIKV residing in the city of Rio de Janeiro, reported to the Information System on Diseases of Notification (SINAN); (ii) sociodemographic data from the census tracts collected by IBGE [38]; and (iii) data on addresses, rainfall, and FHS coverage for the years 2015 and 2016 from Pereira Passos Institute of the Rio de Janeiro Municipal Government [39].

The data from IBGE and Rio de Janeiro Municipal Government are available in electronic databases. However, data recorded in the Information System on Diseases of Notification (SINAN) are available from [State Health Department of Rio de Janeiro]. The authors make the data available upon request and permission of [State Health Department of Rio de Janeiro].

The temporal aggregation of new ZIKV cases was based on the SINAN database in the years 2015 and 2016. Cases were geocoded by residential address on the notifications, using API (Application Programming Interface) from Google Maps [40]. Geocoded ZIKV cases were then aggregated by census tracts, allowing the construction of crude ZIKV cumulative incidence per 1000 inhabitants. To control the incidence rates’ instability effect we used local empirical Bayesian smoothing, weighting the incidence rates of neighboring tracts [41]. We then applied the rates’ logarithmic transformation with Bayesian smoothing to approach them to a normal distribution, thereby establishing the study’s outcome, hereinafter the “ZIKV cumulative incidence”.

Rainfall data were collected from 33 precipitation stations in the city of Rio de Janeiro from November 2015 to February 2016. These data were used to build the variable mean rainfall by census tract via spatial interpolation. Since this information was not measured in all the census tracts, but in 33 precipitation stations in the city of Rio de Janeiro, geostatistical techniques were used to estimate rainfall in areas without precipitation stations. This was used to create a continuous map with the estimated rainfall values for entire city of Rio de Janeiro through simple kriging. This method was chosen since it was expected that the mean monthly rainfall in millimeters from November 2015 to February 2016 would be constant across the surface. Next, the census tract map was superimposed on this map, which allowed calculating the mean rainfall for each tract in the selected period. To perform the spatial interpolation, a 5,000,000 × 5,000,000 grid was defined, since some areas were very small. After spatial rainfall analysis, the model with the best fit was spherical anisotropy effect [42]. The variogram indicated that rainfall distributed differently in all directions. Thus, in the kriging analysis, the anisotropic model was considered. The Figure S2 show 33 precipitation stations in the city of Rio de Janeiro.

Data on coverage by the Family Health Strategy (FHS) defined whether or not the census tract was covered by the FHS.

These databases were then merged, using the census tract number as the identifier field. This allowed a final database with ZIKV cumulative incidence per census tract as the dependent variable (outcome) and sociodemographic data, mean rainfall, and FHS coverage in each census tract as the independent variables.

### Data analysis

We assessed the presence of spatial autocorrelation in the ZIKV cumulative incidence per census tract, using global Moran’s index. The Local Indicator of Spatial Association (LISA) was used to investigate spatial association patterns at the local level, disaggregating the global Moran’s index. LISA classifies the tracts based on the neighborhood matrix, in four groups: high/high, low/low, high/low, low/high. The first two groups correspond to positive associations between a census tract’s incidence rate and the respective neighbors’ rates, while the other two groups represent negative associations [43].

The theoretical model to represent the relations between the ZIKV cumulative incidence and possible associated factors was built from a combination of two conceptual models. “Model I” was proposed by Diderichsen, Augusto, and Perez [44]. The authors summarized a diagram containing possible mechanisms of social inequality in relation to ZIKV infection. These mechanisms were: income, household conditions, vector density, vulnerability, susceptibility, social context, and health policies. “Model II” related the occurrence of natural disasters (earthquakes, floods) to the increase in the mosquito population and consequently to the rise in ZIKV incidence [45]. According to the available data, this study proposed a new model including socioeconomic dimensions, household conditions, and health policies (model I) and rainfall (model II).

**Fig. 2:**
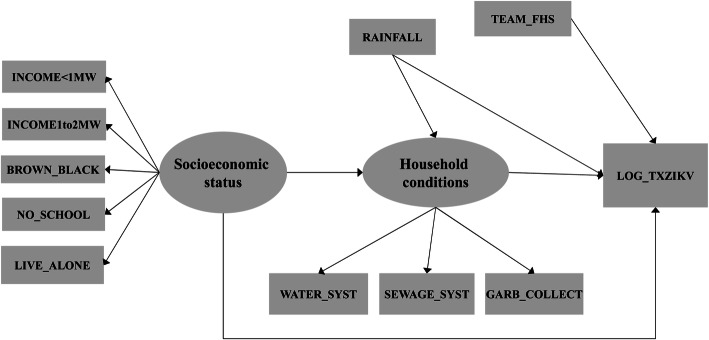
Proposed theoretical model to represent relations between ZIKV incidence and possible associated factors. Source - Owner

Figure 2 presents this study’s proposed model as a causal diagram [46] associated with the Structural Equation Model (SEM) [47]. In this diagram, the ellipses correspond to the construct with two latent variables, i.e., which were not measured (socioeconomic status and household condition). The first latent construct has five indicators represented by the observed variables: INCOME< 1 MW (proportion of households with income less than 1 minimum wage); INCOME1to2MW (proportion of households with income from 1 to 2 minimum wages); BROWN_BLACK as the proportion of households with individuals self-identified as brown or black; LIVE_ALONE (proportion of households with persons living alone); NO_SCHOOL (proportion of illiterates). The latent variable “household conditions” is represented by 3 indicators: WATER_SYST (proportion of households with running water supply); SEWAGE_SYST (proportion of households connected to the public sewage disposal system); GARB_COLLECT (proportion of households with public garbage collection). The variable RAINFALL represents the mean monthly rainfall in millimeters from November 2015 to February 2016; TEAM_FHS shows whether the census tract was covered by Family Health Strategy teams.

One can thus hypothesize that census tracts with heavy social inequality (low income, no schooling, and no family support) can present worse living conditions, also impacting household conditions. Meanwhile, heavier rainfall in a given region an also affect the household, for example, with accumulation of water in mosquito breeding sites. These combined causes may have contributed to the increase in ZIKV cases. In addition, the effect of health policies such as the Family Health Strategy can promote educational activities in the fight against *Aedes aegypti*, which would tend to reduce the number of cases, while also offering greater access to health services, allowing a reduction in underreporting of ZIKV cases.

The analysis excluded 332 census tracts that represented areas with lagoons, forests, green areas, and individuals in institutionalized sites or without population information from the IBGE.

After confirmation of spatial autocorrelation, we assessed the relationship between the outcome (ZIKV cumulative incidence) and the independent variables through Spearman correlation analysis (ρ). Variables with ρ > 0.3 were tested in the model with interaction. The ρ > 0.3 suggests moderate or high correlation [48] . The Spearman correlation matrix showed a statistically significant correlation with the study variables. However, there was strong correlation (indicating possible collinearity) between INCOME< 1 MW and BROWN_BLACK; INCOME< 1 MW and NO_SCHOOL; and BROWN_BLACK and NO_SCHOOL.

The model’s fit was assessed with Ordinary Least Squares (OLS), Spatial Lag Model (SAR), and Spatial Error Model (CAR). The first method includes the traditional linear regression approach, the second incorporates spatial dependence into the dependent variable, and the third method includes the spatial effect jointly in the model’s random component (error) [49]. To choose the model with the best fit, we opted to compare the models according to the highest log-likelihood value and the lowest Akaike information criterion (AIC) value. The spatial dependence term estimates the magnitude of autocorrelation, quantifies the similarly sites in the residual errors [50]. The diagram in Figure S1 shows the path flows for regression analysis. The spatial parameter (ρ) in the CAR model is called “λ”.

Diagnosis of collinearity was performed with the Variance Inflation Factor (VIF) with tolerance values less than 10 [51]. Besides that, spatial stratified heterogeneity (SSH) was evaluated in the study to avoid the possible confounding. The q statistic was used to measure the correlation between ZIKV cumulative incidence and each independent variable. Values of q close to 1 may indicate confounding [52]. Geodetector was utilized for the SSH analysis [53].

All the models’ residuals were assessed with the Moran index to quantify the degree of spatial dependence.

The analyses and map production were performed in the R statistical package, version 3.4.3 [54] and in GeoDa [55].

## Results

### Socioenvironmental factors associated with Zika incidence

A total of 39,331 ZIKV cases were reported in the city of Rio de Janeiro, of which 6536 (16.6%) in 2015 and 32,795 (83.4%) in 2016. The proportion of non- geocoded cases was only 3.4% of the total. The Santa Cruz and Rocinha neighborhoods had the highest proportions of missing data, respectively 10 and 5%. A total of 10,172 census tracts were analyzed, showing high spatial autocorrelation assessed by the Moran index (0.56) and with statistical significance (*p*-value = 0.001).

**Fig. 3:**
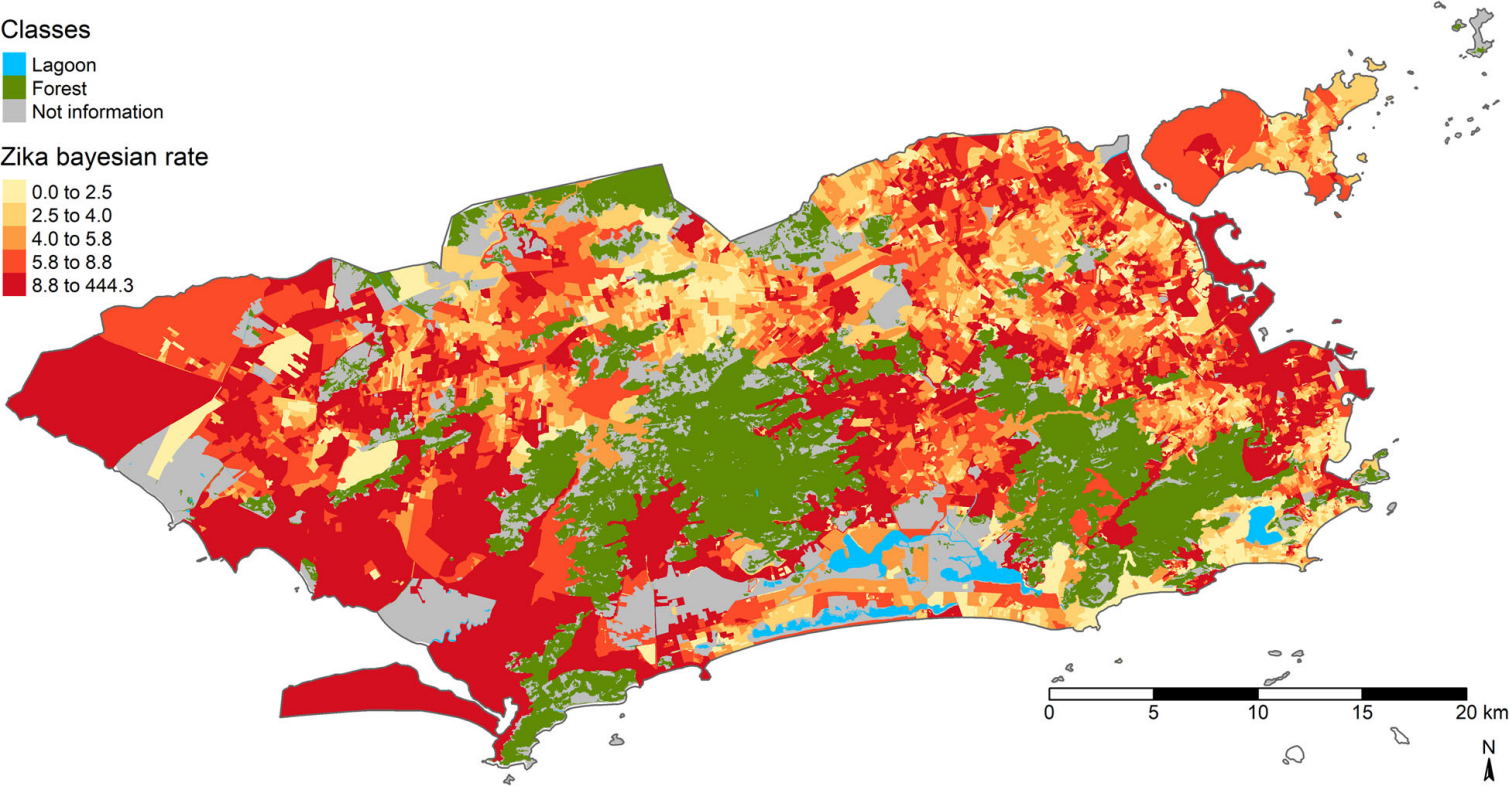
Zika quintile cumulative incidence map. Map created in R software version 3.4.3 by authors. Creative Commons by license IBGE, copyright 2020. Source - Brazilian Institute of Geography and Statistics [38]

Figure 3 shows the distribution of ZIKV cumulative incidence by census tract after Bayesian smoothing. In this figure, the highest incidences are concentrated in the far western area of the city, or PA 5; in the some localities in PA 4; and in localities in PA 3 and PA 1. The gray areas represent the census tracts without population data, the blue areas are lagoons, and the green areas are forests.

**Fig. 4:**
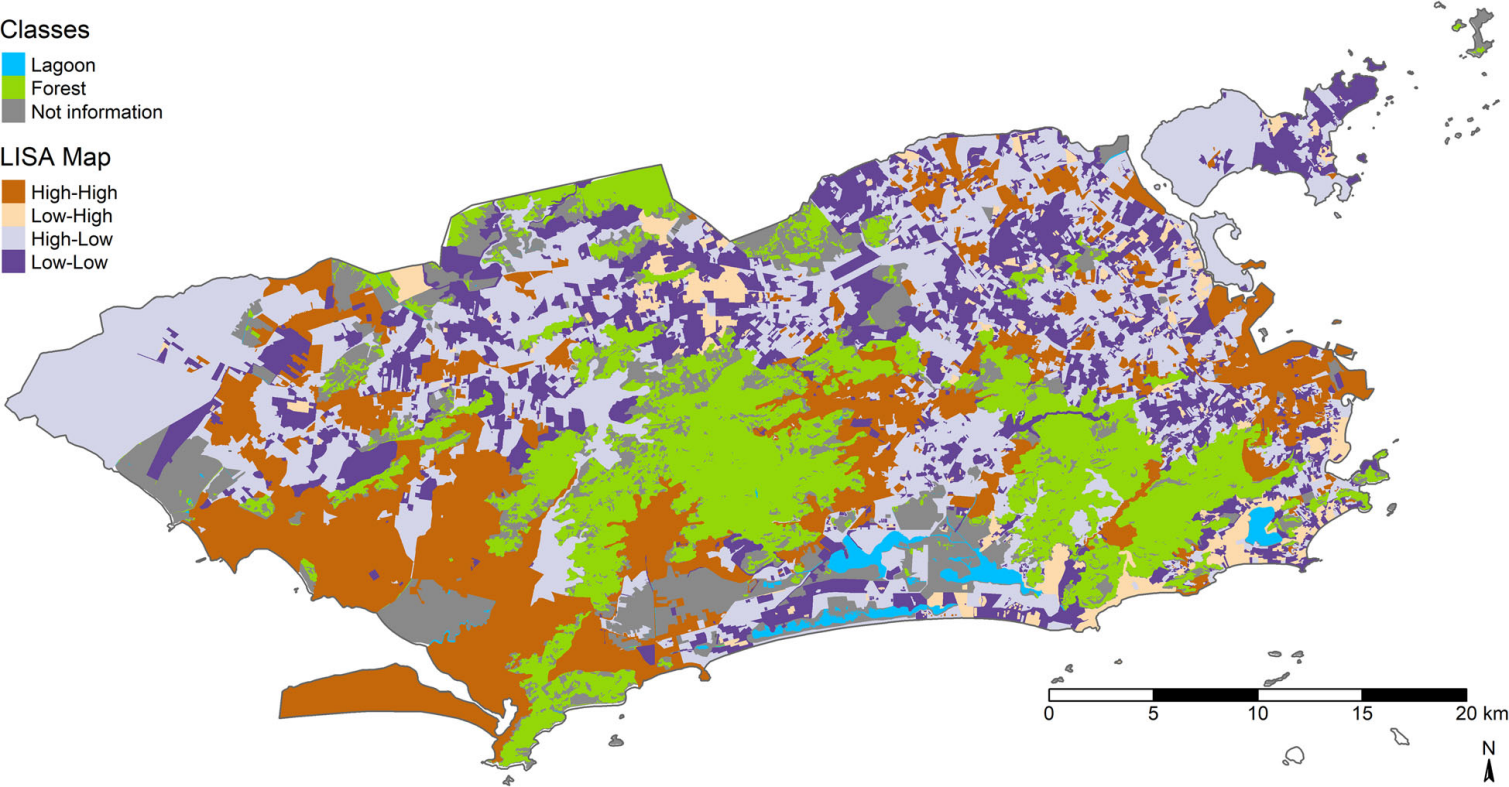
LISA scatter map of Zika cumulative incidence. Map created in R software version 3.4.3 by authors. Creative Commons by license IBGE, copyright 2020. Source - Brazilian Institute of Geography and Statistics [38]

Figure 4 shows the LISA scatter map. The areas with census tracts with high incidence and surrounded by tracts with high rates were concentrated in PA 5, PA 4, localities in PA 2, localities in PA 3, and practically all of PA 1. Meanwhile, areas with census tracts with low ZIKV cumulative incidence and surrounded by tracts that also have low rates are located in the southern portion of PA, localities in the southern portion of PA 4, and localities in PA 5.

**Fig. 5:**
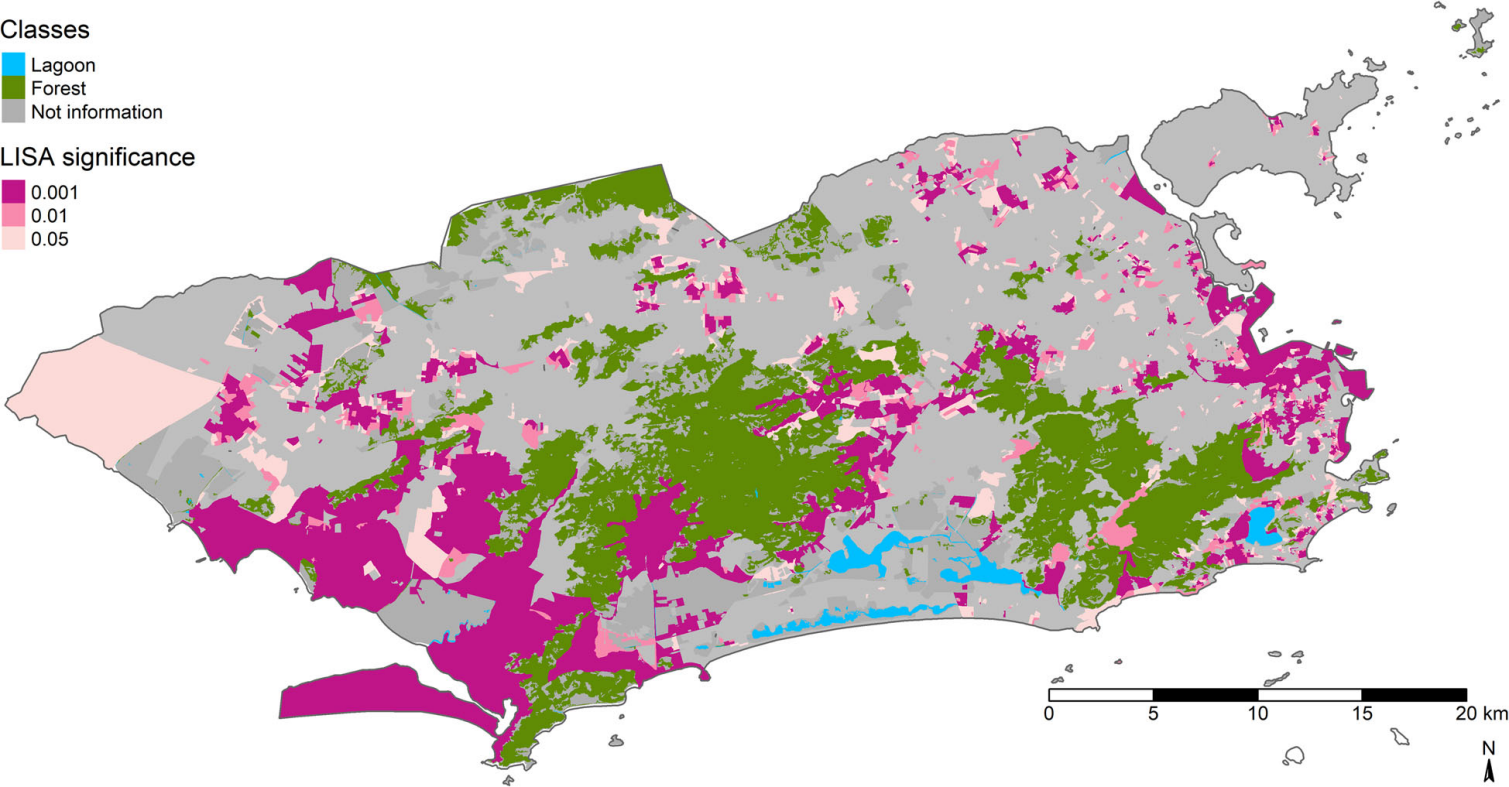
Statistical significance of the local Moran indices of the Zika cumulative incidence. Map created in R software version 3.4.3 by authors. Creative Commons by license IBGE, copyright 2020. Source - Brazilian Institute of Geography and Statistics [38]

Figure 5 shows the areas with statistically significant local Moran indices. Localities classified as high-high and as low-low both corresponded to areas with significant local Moran indices.

As shown in Table 1, the Spearman correlation matrix indicates that the correlation between the log of the ZIKV cumulative incidence and other independent variables was low (less than 0.3). The highest absolute value was for the variable INCOME1to2MW (ρ = 0.223), while the lowest correlation was for TEAM_FHS (ρ = 0.052). All the correlations were statistically significant. There are three high correlations between the independent variables in the matrix, namely INCOME< 1 MW and BROWN_BLACK (ρ = 0.881); INCOME< 1 MW and NO_SCHOOL (ρ = 0.808); BROWN_BLACK and NO_SCHOOL (ρ = 0.756), possibly indicating collinearity.

**Table 1:** Spearman correlation for Zika virus incidence and variables related to socioeconomic status, household conditions, and rainfall

*–*	*INCOME < 1 MW*	*INCOME1to2MW*	*BROWN_BLACK*	*NO_SCHOOL*	*WATER_SYST*	*SEWAGE_SYST*	*GARB_COLLECT*	*LIVE_ALONE*	*RAINFALL*	*TEAM_FHS*
*LOG_TXZIKV*	0.122^a^	0.223^a^	0.099^a^	0.096^a^	−0.148^a^	− 0.192^a^	0.055^a^	− 0.084^a^	− 0.174^a^	0.052^a^
*INCOME < 1 MW*		0.076^a^	0.881^a^	0.808^a^	−0.221^a^	−0.337^a^	− 0.212^a^	−0.205^a^	− 0.320^a^	0.245^a^
*INCOME1to2MW*			0.218^a^	0.061^a^	−0.001	−0.072^a^	0.094^a^	−0.146^a^	− 0.269^a^	−0.059^a^
*BROWN_BLACK*				0.756^a^	−0.199^a^	−0.322^a^	− 0.198^a^	−0.188^a^	− 0.309^a^	0.215^a^
*NO_SCHOOL*					−0.253^a^	−0.369^a^	− 0.270^a^	−0.233^a^	− 0.268^a^	0.183^a^
*WATER_SYST*						0.447^a^	0.211^a^	0.066^a^	0.043^a^	−0.003
*SEWAGE_SYST*							0.198^a^	0.139^a^	0.126^a^	0.028^a^
*GARB_COLLECT*								0.037^a^	−0.033^a^	−0.051^a^
*LIVE_ALONE*									0.225^a^	0.018
*RAINFALL*										−0.011

^a^ level of significance: 0.05

Based on assessment of the regression models by parsimony criteria, goodness-of-fit, and diagnosis of collinearity for the VIF, we present the results of the final regression models for OLS, SAR, and CAR (Table 2). The variables NO_SCHOOL and BROWN_BLACK were removed from the model since they were colinear with income and also showed inverse correlation with the outcome (see Table 1). The variables LIVE_ALONE and RAINFALL did not remain in the final model, based on the statistical significance criterion. Meanwhile, the variable GARB_COLLECT was removed because it showed correlation with the other two variables related to household conditions (WATER_SYST and SEWAGE_SYST), besides displaying asymmetric distribution (Figure S3). The interaction terms evaluated were INCOME< 1 MW versus BROWN_BLACK; INCOME< 1 MW versus NO_SCHOOL; INCOME1to2MW versus BROWN_BLACK; INCOME1to2MW versus NO_SCHOOL. The criterion of biological significance used for these interactions was that poorer people who have less education tend to have a higher risk of infection for ZIKV and other arboviruses.

**Table 2:** Results of regression model, fit indices, and residuals for the Zika cumulative incidence

*Variables*	OLS		SAR		CAR	
	*coefficient*	*p-valor*	*coefficient*	*p-valor*	*coefficient*	*p-valor*
INCOME< 1 MW	0.06	0.010	−0.10	< 0.001	− 0.27	< 0.001
INCOME1to2MW	1.40	< 0.001	0.58	< 0.001	0.44	< 0.001
WATER_SYST	−0.49	< 0.001	−0.19	< 0.001	− 0.27	< 0.001
SEWAGE_SYST	−0.35	< 0.001	− 0.09	< 0.001	−0.09	< 0.001
TEAM_FHS	0.07	< 0.001	0.04	< 0.001	0.04	0.080
R^2^	0.078		0.440		0.438	
Log-likelihood	−10,020.37		−7482.16		− 7495.20	
AIC	20,054.74		14,980.31		15,006.39	
The spatial dependence term			ρ = 0.718		λ = 0.740	
Moran – residual	0.50	0.001	−0.04	0.999	−0.05	0.999

In addition, the interaction terms were not statistically significant in the model.

The variables that remained in the model were those related to income (INCOME< 1 MW and INCOME1to2M) running water and sewage disposal coverage (WATER_SYST and SEWAGE_SYST), and coverage by the Family Health Strategy (TEAM_FHS). The model with the best fit was the SAR model, with a log-likelihood of − 7482.16 and AIC of 14,980.31. In this model, the INCOME< 1 MW variable were negatively associated with higher ZIKV incidence rates. The INCOME1to2M variable was possible risk factors for Zika occurrence in the localities. Variables related to adequate water supply and the existence of public sewage disposal were associated with lower ZIKV incidence rates. The presence of the Family Health Strategy in the census tracts was positively associated with the ZIKV incidence rate.

In the diagnosis of collinearity via VIF, all the values were below 10, indicating absence of collinearity (Table 3).

**Table 3:** VIF values for OLS model

INCOME< 1 MW	1.156
INCOME1to2MW	1.024
WATER_SYST	1.229
SEWAGE_SYST	1.300
TEAM_FHS	1.078

The spatial stratified heterogeneity not found statistically significant association between Zika cumulative incidence and independent variables (Table 4).

**Table 4:** SSH values for independent variable

Variables	q-statistic	*p*-value
INCOME< 1 MW	0.287	< 0.001
INCOME1to2MW	0.227	< 0.001
WATER_SYST	0.008	0.853
SEWAGE_SYST	0.023	< 0.001
TEAM_FHS	0.002	< 0.001

**Fig. 6:**
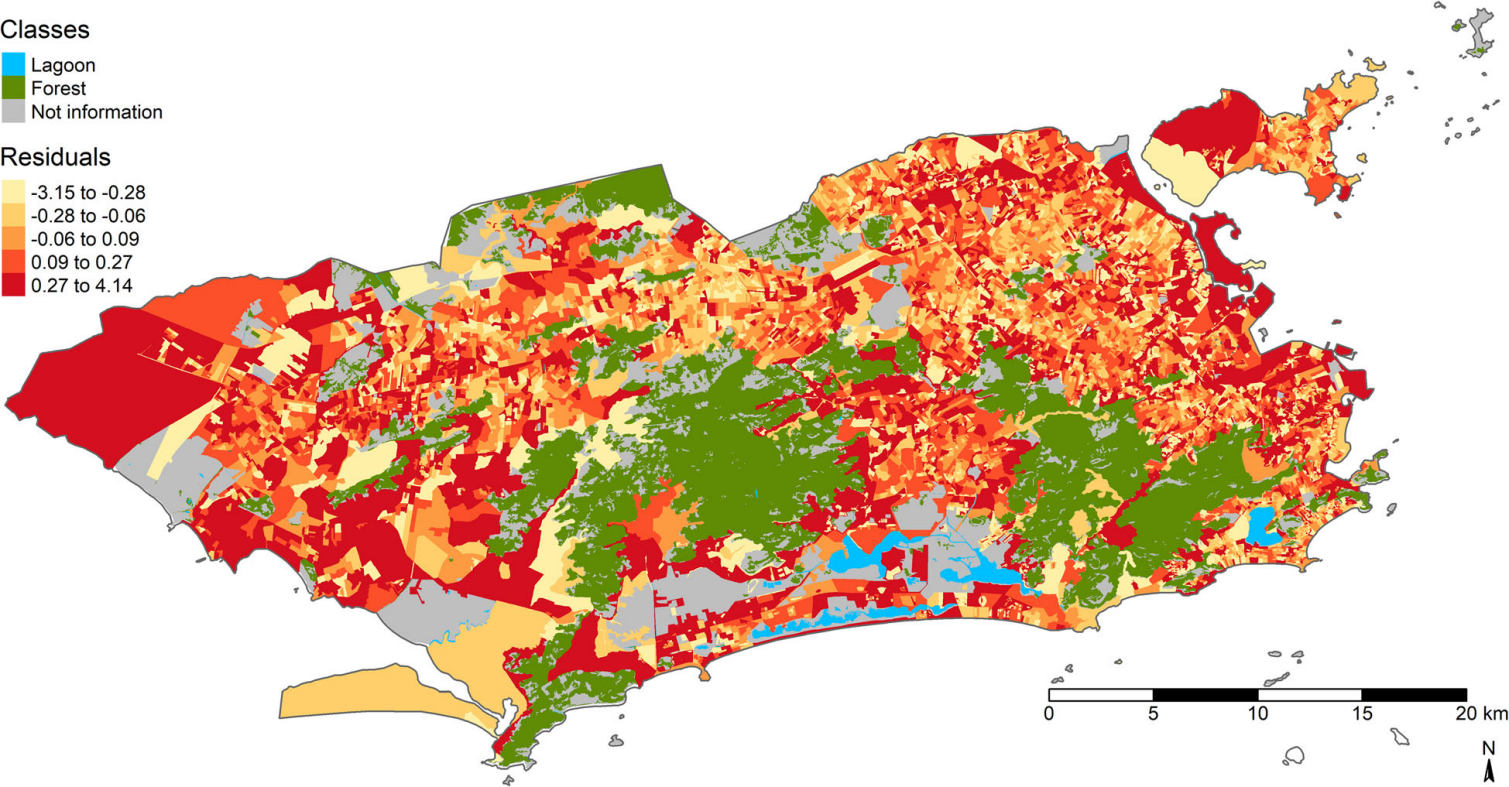
Spatial distribution of residuals in the SAR model. Map created in R software version 3.4.3 by authors. Creative Commons by license IBGE, copyright 2020. Source - Brazilian Institute of Geography and Statistics [38]

The Moran index of the residuals for the SAR model was 0.04 (*p* = 0.999), indicating that spatial dependence was controlled. The Moran index in the OLS model, which does not take spatial dependence into account, was high (0.50) and significant (*p* = 0.001). The Fig. 6 shows the Moran residuals map in the SAR model. Note that the residuals were well distributed across all areas of the city.

## Discussion

In relation to analysis of socioenvironmental factors associated with the ZIKV epidemic in the city of Rio de Janeiro, lower income was associated with higher ZIKV incidence. The study thus demonstrates that less favorable socioeconomic conditions related to income are directly associated with higher ZIKV incidence rates. A study in Salvador, Bahia, Brazil, in 2009 and 2010 showed higher risk of dengue, a disease transmitted by the same mosquito vector, in households with income less than or equal to one minimum wage [56]. In the city of Rio de Janeiro, study suggest that social vulnerability areas can be influencing occurrence of the dengue [57, 58].

The associations found in the current study are valid at the census tracts level and should not be extrapolated directly to the individual level, which would create a risk of ecological bias [59].

The localities with higher coverage of running water and public sewage disposal showed lower ZIKV incidence rates. According to Campos et al. [60], areas more favorable to larval development present worse infrastructure conditions. These two variables combined can indicate lack of environmental sanitation, representing areas with housing that lacks basic infrastructure conditions [61].

Areas with coverage by the Family Health Strategy presented higher ZIKV incidence, indicating that there may be better access to health services and higher notification of cases in these tracts. Kikuti et al. [56] found a decrease in dengue risk in census tracts located farther from health units. However, with the improvement of control methods for the disease and health education activities by healthcare workers, the tendency may be to decrease the ZIKV incidence rates.

This census block study presented some limitations, such as the fact that it did not include the *Aedes aegypti* larval infestation index. Although this indicator was available on the website of the Rio de Janeiro municipal government, it was generalized to the entire study area. In addition, it is not always possible to find a positive association between the larval index and incidence of the diseases, due to difficulties in adequate measurement of the index involving various fieldwork problems, such as closed households, difficulties in access due to public security problems, and even inadequate data collection by health agents. A study in the city of Rio de Janeiro found an inverse association between the Breteau index for *Aedes aegypti* and dengue incidence in 2006 [10]. Other variables that did not enter the model are temperature and relative humidity, which are important factors that influence vector density. However, the range in these variables was very small, so we opted not to include them in the current study (Figures S4 and S5). Rainfall varied more in the city, but it was not significant in the final model. A possible explanation for this would be the use of averages, which could mask the differences between the localities or the rainfall intensity, since more intense rain tends to generate floods and thus drag various breeding sites into the storm drain system. This could also be due to the chosen time window. This study used a 4-month period (November 2015 to February 2016) to estimate mean rainfall. This period was chosen because it coincides with the months with the highest mean rainfall in the city of Rio de Janeiro and the start of the upward curve in reported ZIKV cases [11]. However, tests were performed with other time windows, such as a 6-month period (September 2015 to February 2016), without finding a significant association with the disease.

Another limitation of the study was not to analyze the spatio-temporal dynamics. However, Freitas et al. [62] realized a study with three arboviruses (dengue, chikungunya, and Zika) in the city of Rio de Janeiro in 2015 and 2016. The results show that Zika clusters occurred between November 2015 and May 2016. Furthermore, clusters for all three simultaneous arboviruses included neighborhoods with high population density and low socioeconomic status.

The use of census tracts favored the sociodemographic characterization of these areas, thereby facilitating the construction of indicators. In addition, a census tract tends to display greater homogeneity in the resident population’s characteristics in the tract and greater heterogeneity in relation to the other tracts. The main problem with the use of the census tract is the small population size, potentially generating great instability in the ZIKV cumulative incidence. The choice of the local Bayesian smoothing method aimed to correct possible errors resulting from the fluctuation that these cumulative incidence tend to present in small areas. The method can also correct possible underreporting of the disease, since the incidence in a small area tends to be similar to that of its neighbors.

One possible limitation to the study is methodological. The study’s results, such as the inclusion of interaction terms, the possibility of effect modification by the TEAM_FHS variable, and the socioeconomic vulnerability gradient in the city of Rio de Janeiro may be explained better by other spatial regression models. Local regression models assume that the spatial process is non-stationary, i.e., the coefficients present spatial heterogeneity. Since the amount of observations (number of area data) is large, the non-stationarity hypothesis tends to be confirmed. The local spatial autocorrelation indicators (Fig. 4) revealed different patterns of spatial association in all the areas of the city of Rio de Janeiro [43]. Geographically Weighted Regression (GWR) can thus be used to measure this variability in each of the city’s census tracts. The variables that were removed from the final model by parsimony (BROWN_BLACK, NO_SCHOOL, and GARB_COLLECT’) can thus be explored from the local point of view. Observing the comparison between two extreme groups: census tracts with better socioeconomic status (SES) (low proportions of households with income less than 1 minimum wage, blacks/browns, and illiterates) and worse socioeconomic status (high proportions of households with income less than 1 minimum wage, blacks/browns, and illiterates). The expected results should indicate that census tracts with worse SES would have higher ZIKV rates, while those with better SES would tend to have lower rates of the disease. However, thes Figures S6 and S7 show the existence of extremely poor areas with low ZIKV cumulative incidence and low SES, respectively. Meanwhile, areas with better SES, especially census tracts close to low-income neighborhoods, had higher ZIKV cumulative incidence. This scenario suggests that other determinants not measured in this study may be associated with ZIKV rates in the city of Rio de Janeiro.

## Conclusions

The ZIKV cumulative incidence in the city of Rio de Janeiro in the years 2015 and 2016 was positively associated with census tracts with mean income between 1 and 2 minimum wages and the presence of family health teams. Household conditions related to lower proportions of running water and adequate public sewage disposal also influenced the increase in cases of the disease. However, the results also point to a population group with mean income below 1 minimum wage with a negative impact on ZIKV cumulative incidence. One hypothesis would suggest more underreporting of cases. Other methodological approaches should be considered to investigate possible spatial heterogeneities.

ZIKV is a disease that can cause malformation of the central nervous system, microcephaly among the concepts of mothers who had the virus. For the city, our contributions help to indicate which environmental factors were most associated with a higher risk of the incidence of the disease and, consequently, the risk of pregnant women becoming infected and having the risk of developing fetuses with Congenital Zika syndrome.

## Supplementary Information

**Additional file 1: Figure S1.** Flow diagram of the analysis models. Source – Owner.

**Additional file 2: Figure S2.** Distribution of precipitation stations. Map created in R software version 3.4.3 by authors. Creative Commons by license IBGE, copyright 2020. Source - Brazilian Institute of Geography and Statistics [38].

**Additional file 3: Figure S3.** Distribution of GARB_COLLECT. Figure created in R software version 3.4.3 by authors. Source – Owner.

**Additional file 4: Figure S4.** Annual average temperature. Figure created in R software version 3.4.3 by authors.

**Additional file 5: Figure S5.** Annual average relative humidity. Figure created in R software version 3.4.3 by authors. Source – Owner.

**Additional file 6: Figure S6.** Comparison of ZIKV rates and SES. Map create in R software version 3.4.3 by authors. Creative Commons by license IBGE, copyright 2020. Source - Brazilian Institute of Geography and Statistics [38].

**Additional file 7: Figure S7.** Comparison of FHS Coverage and Low SES. Map create in R software version 3.4.3 by authors. Creative Commons by license IBGE, copyright 2020. Source - Brazilian Institute of Geography and Statistics [38].

## Data Availability

The datasets analyzed during the current study consisted of all confirmed cases of ZIKV. Data recorded in the Information System on Diseases of Notification (SINAN), that supporting the results of this study are available from [State Health Department of Rio de Janeiro]. The data are, however, available by the authors upon reasonable request and with permission of [State Health Department of Rio de Janeiro]. The socioeconomic data from the census tracts of the city of Rio de Janeiro, based from the latest Population Census publicly available in the electronic database by the Brazilian Institute of Geography and Statistics (IBGE), republished from under a Creative Commons by license IBGE, copyright 2020. Addresses, rainfall, and FHS coverage data were collected from Pereira Passos Institute of the Rio de Janeiro Municipal Government, available from electronic database.

## Abbreviations

AIC: Akaike Information Criterion
API: Application Programming Interface
AR: Administrative regions
BROWN_BLACK: Proportion of households with individuals self-identified as brown or black
CAR: Spatial Error Model
CHIKV: Chikungunya virus
DENV: Dengue virus
FHS: Family Health Strategy
GARB_COLLECT: Proportion of households with public garbage collection
IBGE: Brazilian Institute of Geography and Statistics
INCOME< 1 MW: Proportion of households with income less than 1 minimum wage
INCOME1to2MW: Proportion of households with income from 1 to 2 minimum wages
LIVE_ALONE: Proportion of households with persons living alone
NO_SCHOOL: Proportion of illiterates
LISA: Local Indicator of Spatial Association
OLS: Ordinary Least Squares
PA: Planning Areas
RAINFALL: mean monthly rainfall in millimeters from November 2015 to February 2016
SAR: Spatial Lag Model
SEM: Structural Equation Model
SEWAGE_SYST: Proportion of households connected to the public sewage disposal system
SINAN: Information System on Diseases of Notification
TEAM_FHS: Shows whether the census tract was covered by Family Health Strategy teams
USA: United States of America
VIF: Variance Inflation Factor
WATER_SYST: Proportion of households with running water supply
WHO: World Health Organization
ZIKV: Zika virus
