# Supplementary figures and images for: Association between socio-environmental factors, coverage by family health teams, and rainfall in the spatial distribution of Zika virus infection in the city of Rio de Janeiro, Brazil, in 2015 and 2016
Source: BMC Public Health. 2021 Jun 23;21:1199. doi: 10.1186/s12889-021-11249-y (PMC8220830; doi:10.1186/s12889-021-11249-y)

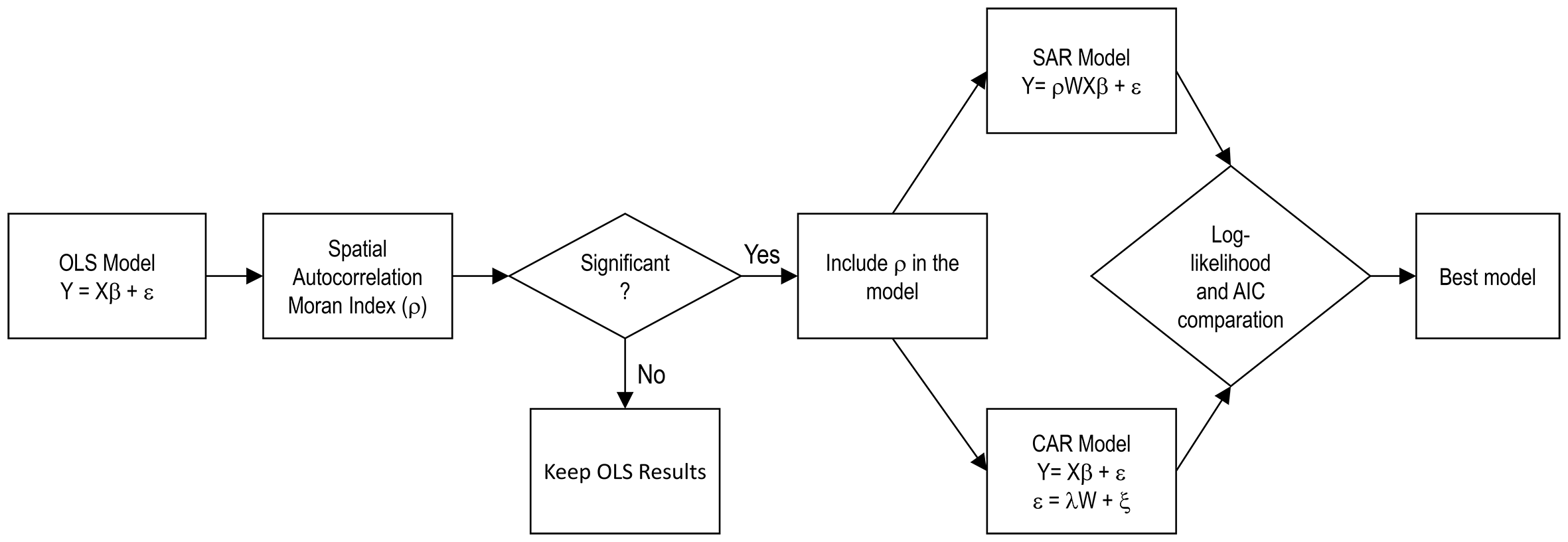

Supplement: Supplementary file 1 — Additional file 1: Figure S1. Flow diagram of the analysis models. Source – Owner. [file 12889_2021_11249_MOESM1_ESM.png]

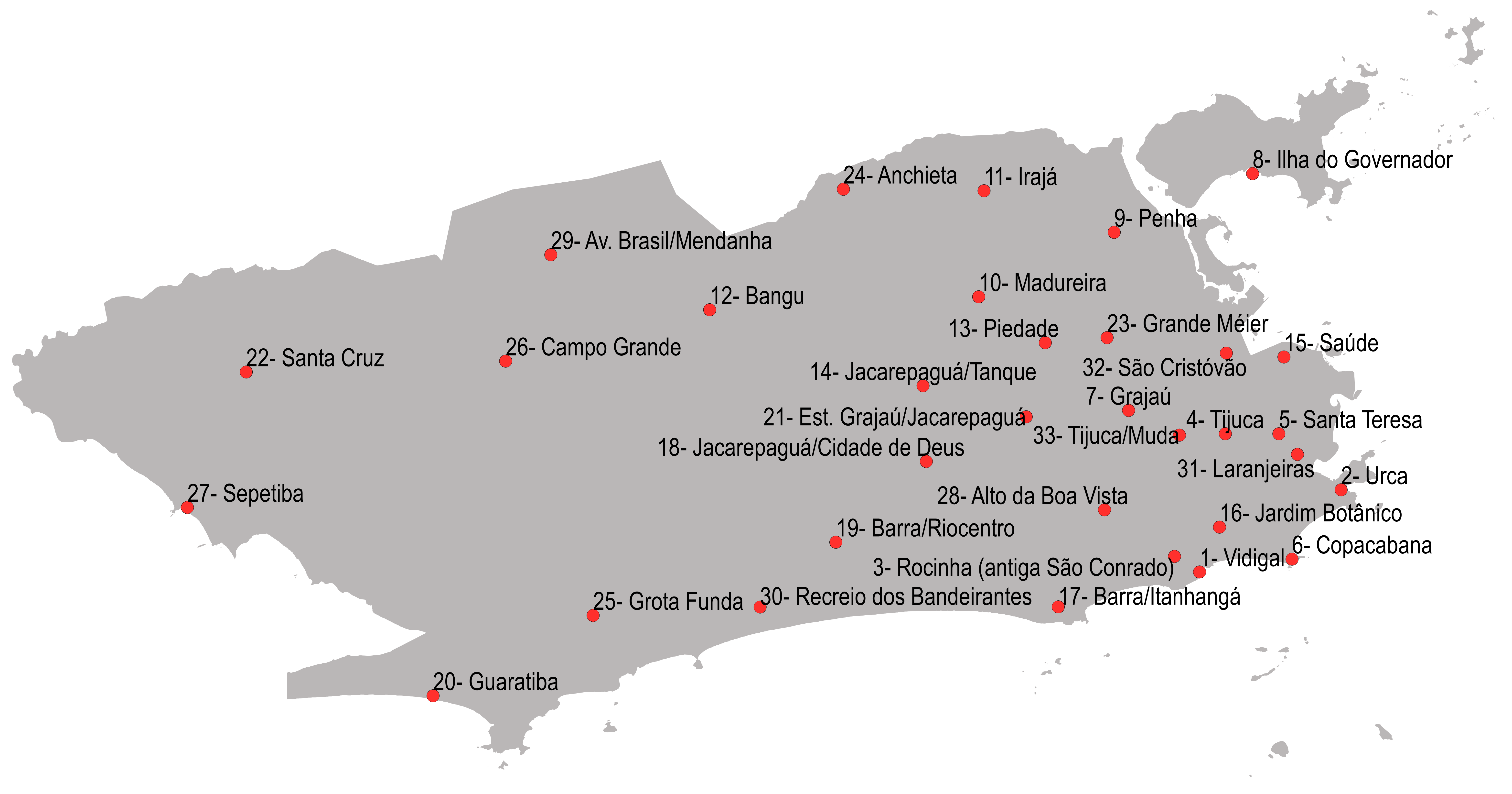

Supplement: Supplementary file 2 — Additional file 2: Figure S2. Distribution of precipitation stations. Map created in R software version 3.4.3 by authors. Creative Commons by license IBGE, copyright 2020. Source - Brazilian Institute of Geography and Statistics [38]. [file 12889_2021_11249_MOESM2_ESM.png]

Distribution of GARB\_COLLECT

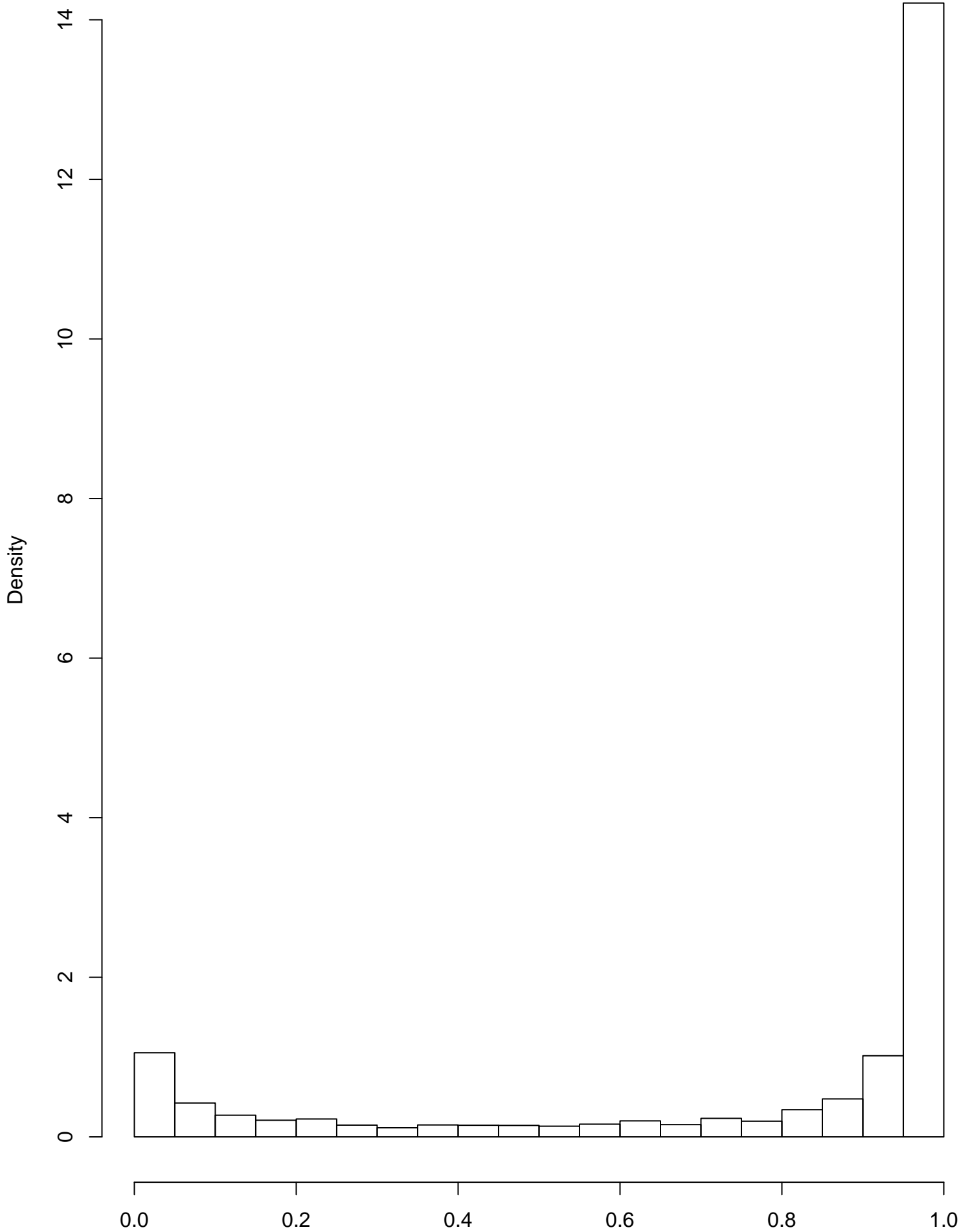

Supplement: Supplementary file 3 — Additional file 3: Figure S3. Distribution of GARB_COLLECT. Figure created in R software version 3.4.3 by authors. Source – Owner. [file 12889_2021_11249_MOESM3_ESM.pdf]

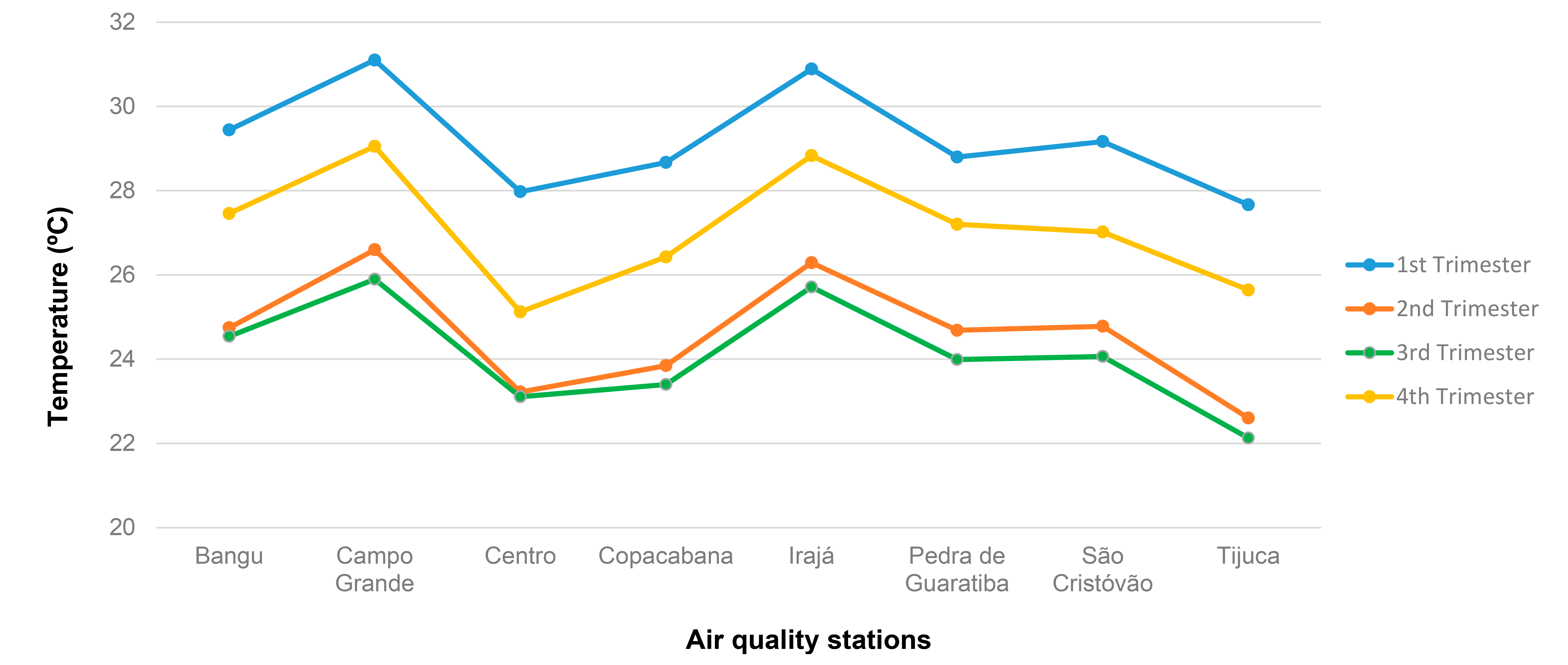

Supplement: Supplementary file 4 — Additional file 4: Figure S4. Annual average temperature. Figure created in R software version 3.4.3 by authors. [file 12889_2021_11249_MOESM4_ESM.png]

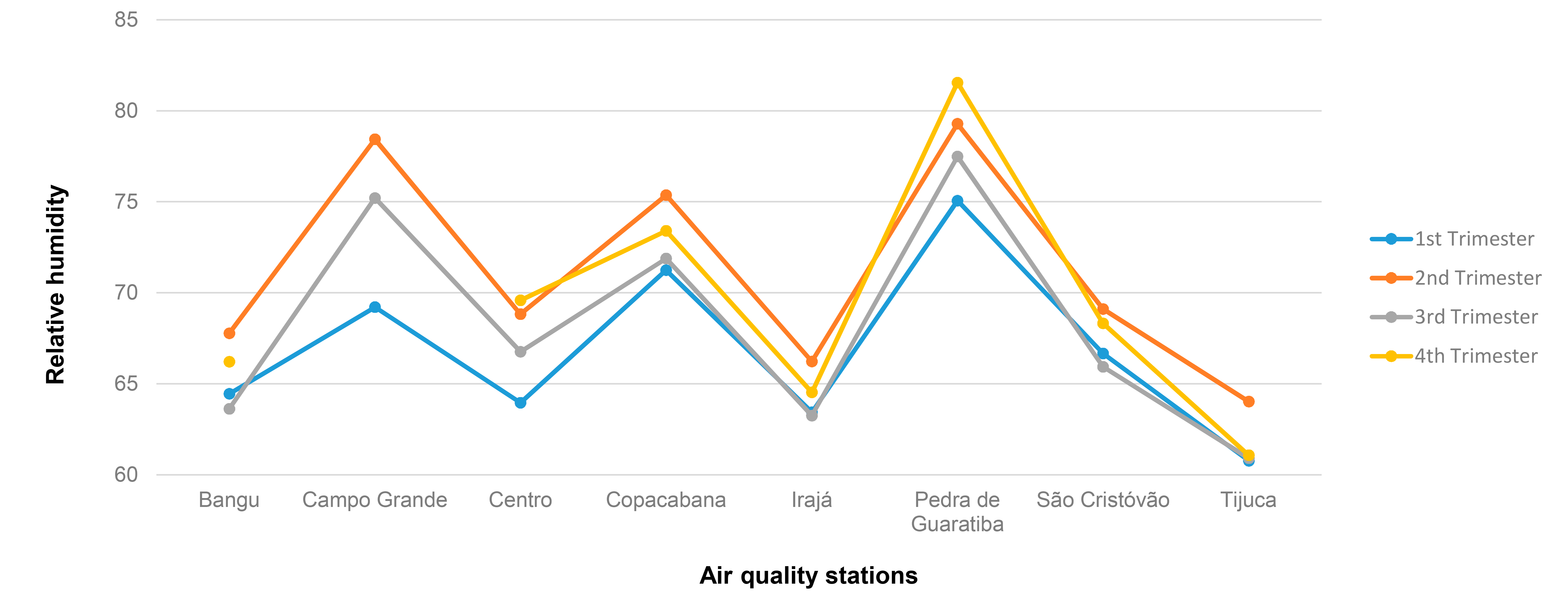

Supplement: Supplementary file 5 — Additional file 5: Figure S5. Annual average relative humidity. Figure created in R software version 3.4.3 by authors. Source – Owner. [file 12889_2021_11249_MOESM5_ESM.png]

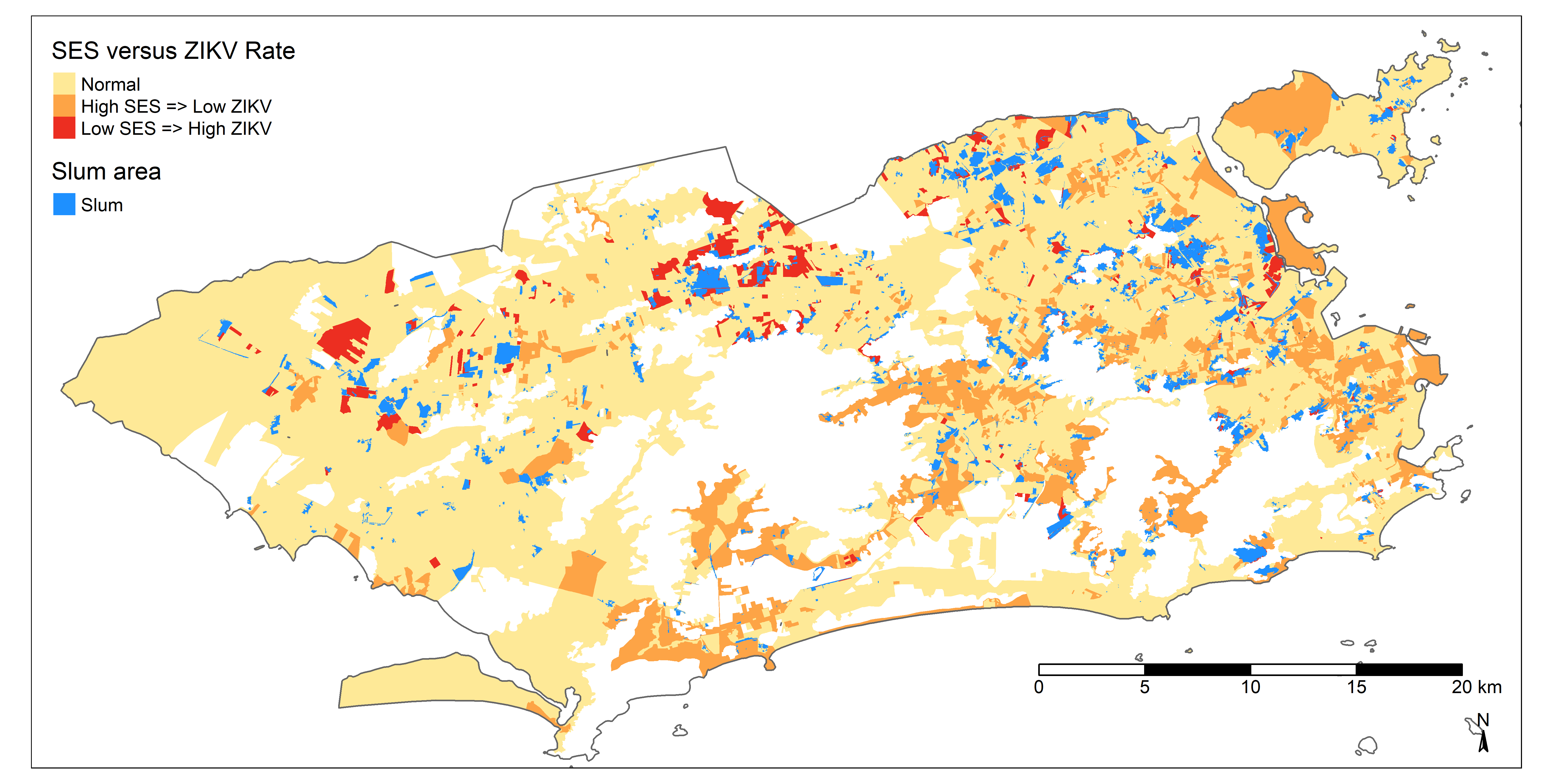

Supplement: Supplementary file 6 — Additional file 6: Figure S6. Comparison of ZIKV rates and SES. Map create in R software version 3.4.3 by authors. Creative Commons by license IBGE, copyright 2020. Source - Brazilian Institute of Geography and Statistics [38]. [file 12889_2021_11249_MOESM6_ESM.png]

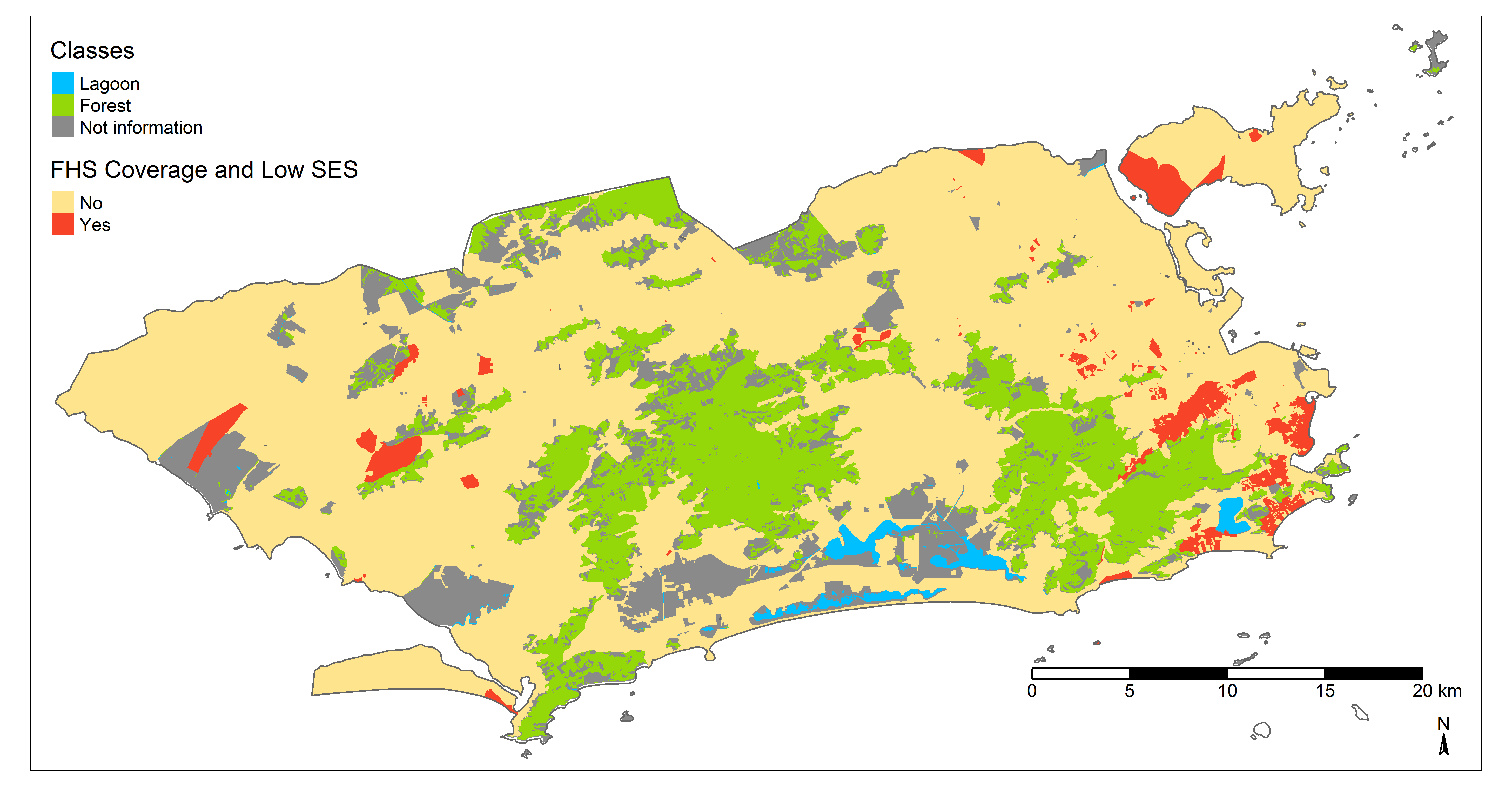

Supplement: Supplementary file 7 — Additional file 7: Figure S7. Comparison of FHS Coverage and Low SES. Map create in R software version 3.4.3 by authors. Creative Commons by license IBGE, copyright 2020. Source - Brazilian Institute of Geography and Statistics [38]. [file 12889_2021_11249_MOESM7_ESM.png]
